# Proceedings of the Cincinnati Academy of Medicine

**Published:** 1865

**Authors:** C. P. Wilson

**Affiliations:** Hall of Academy of Medicine


### PROCEEDINGS OF THE CINCINNATI ACADEMY OF MEDICINE.

Reported by C. P. Wilson, M. D., Secretary.

Hall of Academy of Medicine,
Monday Evening, Jan. 23, 1864.

*Death from Chloroform.*—Dr. Wood remarked, in regard to the death from chloroform at the Commercial Hospital, that it was not necessary to say anything as to the general condition of the patient, or the post mortem appearance, as the report of Dr. Taylor sufficiently covered these points. On that Saturday's clinic there were several operations to be performed, both minor and capital, those cases were operated on before the fatal case; all these, in fact all operated on that day, took chloroform from the same bottle, and to all administered by the same person; one patient, just preceding the case with fatal termination, struggled violently and was much more affected than the one that died, though all unpleasant symptoms passed off after the struggling ceased. Next came this case to which the anæsthetic from the same bottle, same cloth, and in about the same quantities was administered. The cloth used at the Hospital is a piece of muslin folded upon itself several times, and covered with a piece of oiled silk; how long used there or by whom introduced he did not know, but supposed it was a matter of economy to prevent the waste of chloroform from its rapid evaporation, had never used this kind in his own private practice, but generally a handkerchief or thin piece of muslin or linen. The same cloth was used in all these cases. Now the error—if any error there was, and he was not inclined to attribute such to any one—was in the almost entire exclusion of the atmospheric air, from the lapping of the cloth, by which the patient got no air but all the time pure vapor of chloroform; the anæsthetic was given and the man went under it kindly with no bad symptoms and his pulse good; the operation was performed, after which, while speaking to the class, he accidently looked around and saw the man gasp and that he was suffocating, he immediately withdrew the tongue and the man breathed; waiting until he had drawn ten or more inspirations he again turned to the class remarking that such circumstances were not unusual, and that they must always be on their guard for them. After this he again turned his attention to the man and saw that he was not breathing and was dead. Artificial respiration and other means were used, but did no good. When he turned the second time there was no pulse at the wrist, nor could he feel the heart pulsate; he thought the heart ceased beating at the same time with the respiration, and that neither one had precedence of the other. As to the cause of death, the post-mortem reveals nothing, with this exception, that the blood of a person under chloroform is driven from the periphery to the centre, from the meninges to the brain, and that there is great congestion of the nervous system of the head, the nervous blood being driven up to the brain.

He had always thought chloroform a dangerous article, and never was certain while operating but that the patient might die from it; he had used it since its introduction but always gave it with fear. Chloroform carried the blood to the great nervous centre, overpowering the brain, the nervous and the muscular system, and the heart. The reasons why all cases do not terminate fatally is that we can produce anesthesia without overcoming the power of the heart, and the involuntary muscles, and that when the anesthesia is carried so far as to subdue the involuntary muscular system, death will always follow. Any one who has operated about the rectum and certain other portions of the body knows that the involuntary muscles are the last to yield, and long after the voluntary muscular system has yielded. In this case the man inhaled enough to overcome the involuntary system, and so died; though there was no doubt there was some peculiarity or idiosyncrasy connected with the case, but what it was he could not say.

As to the pathological changes found after death they were not the result of chloroform; that he was certain he had given chloroform where there was a much worse condition of the organs of the body, even where effusion into the lungs, and also in cases of paralysis. As to the cases of amputations at the hip joint in the Crimea, spoken of by Dr. Buckner, he thought they should not be charged to chloroform, but rather to shock and of hemorrhage. He was satisfied that he had given chloroform in some cases where without it they would have sunk under the operation, but by the aid of the anæsthetic had stood the operation bravely, and contrary to expectation had done nicely afterwards. Thought chloroform a blessing to humanity, and that we should not be discouraged even though we did lose a case once in a while; but one thing, he would never again let a patient take it with so great exclusion of atmosphereic air. As to ether and chloroform, even now after this death he had no more fear of the latter than of the former. After the introduction of ether and chloroform, he first used ether alone, but finding that he could not bring his patient to such a state of unconsciousness as to operate carefully, he next tried (as introduced by Dr. Reuben Mussey) a mixture of ether and chloroform; two parts of the former to one of the latter, with more satisfactory results. Ether he thought produced more struggling, restlessness and spasmodic excitement than chloroform; had seen cases where even the above mixture would not quiet, so used chloroform alone.

One fact he had noticed that persons accustomed to using liquor did not come under the influence of chloroform so easily and speedily as temperate persons.

The only case where he had ever been alarmed, with the exception of the recent case at the Hospital, was one where he used the mixture before stated, a young lady, full of good country blood came to him with strabismus, he did not wish to give an anæsthetic for so simple a matter, but she being unwilling to undergo the operation without it, he without any preparation of her system gave her the mixture and operated; after the operation she suddenly ceased to breathe, and for fully ten minutes remained so before she was restored. His belief was that any agent that will produce anæsthesia is dangerous, and if carried far enough will produce death; that of all the anæsthetics, ether, chloroform, nitrous oxide, &c., no one of them is more dangerous than another.

Even though he had lost one patient, yet he was not discouraged, nor had he lost confidence in chloroform; and on the same Saturday administered it to another case just after one had died. It was an amputation of both feet; the chloroform was from the same bottle and given in the same way; the patient was under it much longer than the one preceding him; yet he did well and is now recovering nicely. Before administering chloroform he always prepared the system of his patients. As said before his theory is that under chloroform the blood is rapidly driven to the head and great nervous centre, as shown by the flushed face and turgid condition of the jugular veins, also that there is a prevention of the return of the blood from the brain, which does not pass off till the effects of the chloroform have disappeared; therefore to derive from the head and prevent this congested condition as much as possible he was always in the habit of purging his patient the night before; again, he did not allow the person to eat the meal preceding the operation—in the morning no breakfast, or in the afternoon no dinner and a very light breakfast—he never would give it on a full stomach. This perhaps seems at variance with the idea of giving stimulents just before the anæsthetic, but he did not believe in it, and unless the patient was very feeble or had been sick for a long time, or as sometimes happens comes to the table under great nervous restlessness he would not allow any stimulents whatever, and never would grant it if the patient is plethoric; if a patient is much reduced from a long illness he bears the chloroform much better than a strong plethoric person; that the reported cases of death from chloroform are generally in strong persons, in general good condition, such as have met with an accident, gun shot wound or something of that nature, and not in those who have been in bed for many weeks and been much reduced or emaciated. Not long since he performed an extensive operation on a woman whose pulse at the time of operation was 150, very feeble and almost imperceptible; she had been wasted by peritonitis and had considerable effusion around a large ovarian tumor; he drew from her a large quantity of water and then removed the tumor; under the chloroform her pulse rose and maintained itself through the operation; no depression resulted from the drug though she died in some four weeks afterwards as expected.

Another fact, where persons have died from chloroform, the fatal result is not determined by the quantity of the anæsthetic used, nor the time the patient is kept under its influence, but death very often happens where a small quantity only is used; for instance a drachm (ʒi.) and the person under it a very short time.

At the previous meeting of the Academy on the evening of the 16th the following account of the post mortem appearances was detailed by Dr. Taylor:

Record of post mortem examination of Henry V, 27 hours after death.

Large frame, corpulent men, apparently healthy. Post mortem rigidity well marked. On removing the body to the table a small quantity of dark colored thin fluid with but little froth flowed from the nostrils. The fluid had the usual odor of the discharge from the nostrils after death. Suggillation of depending parts of body, and over anterior surface of chest of lighter color than usual. The scalp was very full of blood which flowed freely from the incision. Dura Mater much congested, serum effused beneath arachnoid, and deposits of lymph on both its surfaces. The surface of medulla oblongata much injected; fine threads of lymph passing from lateral and under surfaces of medulla oblongata to the cerebellum. In right hemisphere of cerebrum the puncta vasculosa were more numerous than usual; a small quantity of bloody fluid in the lateral ventricles; threads of lymph passed from the floor to the roof of each ventricle; the choroid plexuses were pale and adherent throughout their entire length; the veins of the ventricles were very full of blood; the velum interposition was covered by firmly adherent lymph. There was considerable fat on the external surface of the pericardium. On the upper and right portion of inner surface of pericardium were numerous patches of soft coagulated lymph; there was rather more than the usual amount of fat on the wall of the heart, but the muscular structure was normal; the cavities were all empty; valves healthy; the right pleural surfaces firmly adherent throughout most of their extent, and the lobes of right lung united by firm false membrane; the lobes of the left lung were also adherent, but the plural surfaces free. The epiglottis and mucous membrane of larynx dusky color; some small firm white deposites beneath mucous membrane of larynx; lungs crepitant only in lower portions; the entire upper lobes of both lungs splenified, the same condition to a less degree in the lower and posterior portions; the lower anterior portions were emphysematious; in lower portion of right lung a small cretaceous mass was found. The blood was thin and dark colored, no coagula were found. There was no odor of chloroform exhaled from the body.

Dr. McIlvaine said that sixteen years ago the 23d of next February the first accident occured from the use of chloroform in the celebrated Simmons case, and that in this it was said by Malgagine that the woman did not die from chloroform but was choaked to death; her blood exhibiting the same characteristics as found in a dog after fatal choking. He was glad a post mortem examination had been made, for if it had not, it would have been said the man died of heart disease—whereby from the examination there was no evidence of it—the heart and the valves being healthy. Dr. McIlvaine referred to a case of Dr. Mussey's where the heart's action ceased, and life was almost extinct, but was restored by drawing forth the tongue, using artificial respiration, &c.; and he thought if in this case the tongue had been drawn from the throat and held there by piercing it with a needle armed with a thread, that the patient might not have died. Then he did not think chloroform had anything to do with the death, and that the views of Malgagine, in the Simmons case, were right, and were applicable here. He said no article of the materia medica was administered so often with so few bad results, and did not think the anæsthetic the cause of death.

Dr. Carroll said that he knew of two fatal cases from the use of chloroform in the summer in the hands of quacks.

Dr. Mussey thought these cases should be made known and put on the records of the Academy. He thought chloroform inimical to life and had so expressed his views to the students at the Commercial Hospital only a short time ago. He did not like it, and often thought he would never use it again. In one case he was preparing to tie the carotid—during the administration of the anæsthetic—(a combination of chloroform and ether) the pulse flagged, and heart pulsated faintly, so that they were compelled to desist from the further use of the anæsthetic; when the pulse came up, at a little interval, at the direction of his father, (Dr. Mussey, Sr.,) they re-administered it, but on arriving at a certain point the same symptoms occurred, and they were again obliged to stop; and so for a third time with the same result, compelling him to desist entirely from the operation for that day. Ten weeks afterwards he operated on this same man, tying the carotid and removing the mass, and the man sunk under it. He believed the vitality of the patient was impaired by the previous administration of chloroform, and that the result was somewhat influenced by it; for certainly the man's general health and powers of endurance were not as they had been before the anæsthetic was used.

In another case, of impassable stricture of the urethra with perineal fistula, for four successive times, they brought the man to that point when a rigid spasm came up, so that they had to suspend the operation. The man not being willing to undergo the operation without chloroform, is still about, having had many fistulæ. Dr. M. thought chloroform more apt to produce these effects than ether, which helps to sustain the action of the heart, while chloroform from its sedative powers depresses it.

On being answered as to the time artificial respiration was kept up—Dr. M. said in the case alluded to by one of the members, he had almost given up the artificial respiration, and at last thrust his finger into the glottis without success; again produced artificial respiration, for awhile ceasing, to make a thrust and a second thrust into the glottis, which was followed by a spasm of that organ; then he continued the artificial respiration till the man breathed freely and naturally. For 7½ minutes there was no voluntary respiration or action of the heart. The recent case at the Hospital was a sad and an important one for us to draw instruction from, and to reflect upon as to whether it was the fault of the agent or a mechanical one. The latter in his opinion generally being the first cause of trouble.

Dr. Fries said he had given chloroform and ether separately, and the two mixed, a great many times; for the last six or seven years he had used nothing but pure chloroform. The only alarming case he had ever seen was fourteen years ago in a case of lithotomy; just as he was about to extract the stone, under the use of chloroform and ether equal parts, the breathing suspended; but fortunately a gentleman of highly excitable and nervous temperament (Dr. Roelker) was present and he quickly drew forth the tongue and respiration returned. He had seen great distress and irregularity of breathing, but generally it was in those cases where the two anæsthetics were mixed; he would not use ether because of the great distress it causes patients. Dr. F. said there were certain precautionary measures which he always regarded, namely, never allowing the administration of the chloroform without first preceding it by a full dose of some stimulent; never giving it except in the horizontal position; also the more rapidly you produce anæsthesia in your patient, the more likely you are to have a safe result; if you give it slowly, in small quantities at a time, frequently removing the cloth to give the patient air, fatal issues will occur, the blood becoming most thoroughly impregnated with it by this tedious process. The handkerchief should be held concave over the mouth, in such a position as to allow the free passage of air from above downward, and he was satisfied you would bring your patient under full anæsthesia in half the time and with half the quantity of the agent by compelling him to inhale it through the mouth, rather than through the nostrils. He had experimented upon himself to the state of unconsciousness four different times by merely dipping the point of a handkerchief in chloroform and placing it between the teeth.

Dr. Richardson was somewhat surprised at some of the remarks of the gentleman, (Dr. Fries), for while he knew of no death from ether, he knew of several from unmixed chloroform. He proceeded to speak at some length of the details of a case some years ago on Vine street in this city, in which a heroic operation was performed on the face, in which case he thought if the patient had died of shock or loss of blood, she should have died on the table, and she died too soon to be from the secondary effects; in his opinion she died from the effects of chloroform. In another case, a patient of his a healthy woman with vesico vaginal fistula, he gave a mixture of chloroform and ether, it was a protracted operation, and during it would frequently permit the woman to return almost to consciousness, and then again etherize her. After the operation she had considerable difficulty of breathing, so that he was considerably alarmed, and visited her again that night in view of this trouble. Her lips were livid, face palad, and breathing asthmatic; it was two or three days before the bad symptoms passed off. In another case he gave it to a gentleman for the purpose of having teeth extracted; he gave it continuously, but at a certain point the man became rigid and he was obliged to desist. He repeated the trial two or three times with the same result; thinking the whole trouble arose from nervous excitement, he directed the man to eat a light breakfast the next morning, and no dinner and repeat the effort at anæsthesia. He gave him a dose of brandy and commenced the use of the chloroform, with precisely the same result as before. He thought there was no denying the fact that a large number of deaths have resulted from the use of chloroform.

Dr. Mussey said he knew of another death from chloroform in the army; a healthy young man of the 18th Regulars, for some trouble in his hand was operated on; chloroform was administered and the man died. The Asst. Surg. who operated attributed the death to the negligence of the person who administered the anæsthetic.

Dr. Fries thought many of the cases lost were owing to the negligence of the person administering, and to the neglect of proper precautions. Sometimes the person administering the chloroform becomes absorbed in the operation, and the patient already chloroformed, continues to inhale the anæsthetic beyond necessity, and of course safety. He had given chloroform in several cases of midwifery practice with the happiest results.

Dr. Bruenn spoke in favor of chloroform—citing the conclusions arrived at by Dr. Stone, of New Orleans, confirming the opinions of Snow, that chloroform is in itself very dangerous in the hands of those who do not know how to employ it.—*Western Lancet.*

